# Family Functioning and Psychosocial Burden in Caregivers of Children with Sickle Cell Disease in a Tertiary Centre in North-Central Nigeria

**DOI:** 10.4314/ejhs.v34i6.13

**Published:** 2024-11

**Authors:** Mohammed Abdulkadir, M Alabi Kola, A Oyeleke Oyeronke, N Alabi Anthonia, O Ademola Christy, R Yusuf Adebayo, A Obalowu Ismaila

**Affiliations:** 1 Department of Family Medicine, Kwara State University Teaching Hospital, Ilorin, Nigeria; 2 Department of Family Medicine, University of Ilorin Teaching hospital, Nigeria

**Keywords:** caregivers, family functioning, psychosocial burden, sickle cell disease

## Abstract

**Background:**

Sickle cell disease (SCD) is a chronic genetic disorder that has significant psychosocial consequences for both patients and their families. This study aimed to investigate family functioning and the psychosocial burden experienced by caregivers of children with SCD.

**Methods:**

This descriptive, cross-sectional study involved 170 caregivers of children with SCD, selected through systematic random sampling. Data were collected using both structured and semi-structured questionnaires. Family functioning was assessed using the Family APGAR (Adaptation, Partnership, Growth, Affectation, Resolve) while the psychosocial burden was evaluated using the Sickle Cell Disease Burden Instrument (SCDBI). Statistical analysis was performed using the Statistical Package for Social Sciences (SPSS) version 24.

**Results:**

The mean age of the participants was 34.7 years, and 83% were female. The majority of caregivers reported a high level of family functioning. However, significant psychosocial burden was observed in areas such as finances, disruption of routine family activities, and the caregivers' coping abilities. The impact on family interactions was minimal.

**Conclusion:**

Most caregivers in this study demonstrated good family functioning. Although caring for a child with sickle cell disease imposed a significant psychosocial burden, the negative impact on family interactions was minimal.

## Introduction

Sickle cell disease (SCD) is one of the most common genetic disorders globally, affecting up to 100 million people, predominantly among Black populations in Africa, Europe, America, Arabia, and those of Asian ancestry. Nigeria has the largest population of people living with SCD (1). SCD is a group of inherited disorders caused by abnormalities in the hemoglobin (Hb) gene. Sickle cell anemia, in which only Hb S is produced, is the most severe and common form (2). Hemoglobin S results from a single base pair change at the 6th codon of the β-globin gene, which encodes valine instead of glutamine at the 6th position of the β-globin molecule (3).

Family functioning refers to how family members interact, react to, and treat one another. It includes variables such as communication styles, traditions, roles, boundaries, and levels of flexibility, adaptation, and resilience (4). The family plays a vital role in mediating and moderating the effects of determinants on health outcomes. A person's health can affect family members' health, while the family environment can influence individual health outcomes (5).

A functional family meets the needs of all its members. High levels of family expressiveness and support, and low levels of family conflict, are linked to improved adjustment among healthy siblings of children with SCD (6). Literature supports a relationship between family functioning and child outcomes, particularly in the adaptation of children with SCD (7).

SCD has both physiological and psychological complications (8). The psychosocial burden refers to a wide range of stressors, including psychological distress, behavioral difficulties, and issues related to relationships and social functioning (9). The psychological impact on caregivers of children with SCD is also significant and is often referred to as a “burden” (10). This burden can be classified into objective and subjective categories. Objective burden includes the daily management of the illness, its impact on other aspects of life, and financial consequences, while subjective burden refers to the emotional distress caregivers experience in caring for their ill child (10).

Research on family functioning in caregivers of children with SCD and other chronic conditions is lacking in this region, despite the high burden of the disease in Nigeria. Most studies on the psychosocial challenges of SCD have been conducted in developed countries. In low- and middle-income countries, particularly in Sub-Saharan Africa, there is disproportionately less research on the psychosocial aspects of SCD. In our environment, family systems are typically close-knit, and the family unit exerts a significant psychosocial influence on the health of its members. This study aims to explore the potential ameliorating effects of family functioning on chronic diseases like SCD, and to guide primary care physicians in the optimal management of the disease burden. It will also contribute to the growing body of knowledge on the disease in this region.

The objectives of this study were to assess family functioning in the families of children with SCD and to evaluate the psychosocial burden experienced by caregivers.

## Materials and Methods

This hospital-based descriptive cross-sectional study was conducted at the Paediatric Sickle Cell Clinic of the University of Ilorin Teaching Hospital. It involved 170 consenting caregivers of children with SCD who were in stable condition and met the inclusion criteria. The sample size was determined using Fisher's statistical formula for health studies (11).

Caregivers were selected using systematic random sampling. The SCD clinic operates once a week, and approximately 560 caregivers were seen over a three-month period. On each clinic day, around 40 caregivers attended, and a sampling interval of 3 was used. Every third caregiver was selected on a first-come, first-served basis until the desired sample size was achieved.

To be eligible, a caregiver had to be an adult (over 18 years) who had lived with the child for at least one year and was involved in the child's intimate care. Caregivers of children diagnosed with SCD less than a year ago, caregivers with chronic diseases, and those with children who had concomitant medical problems (e.g., asthma) were excluded. The study was approved by the hospital's Ethical Review Committee. The financial costs of the research were borne solely by the authors.

**Data collection**: Data were collected using structured and semi-structured questionnaires administered by the researcher. Family functioning was assessed using the Family APGAR, which includes five parameters: Adaptability, Partnership, Growth, Affection, and Resolve. The response options were on a 3-point scale, ranging from 0 (hardly ever) to 2 (almost always). Family APGAR scores were interpreted as follows: 7-10 (highly functional family), 4-6 (moderately dysfunctional family), and 0-3 (severely dysfunctional family). Family APGAR has been validated and used in previous studies in Nigeria (12).

The psychosocial burden was assessed using the Sickle Cell Disease Burden Instrument (SCDBI), which was initially validated by Ohaeri and Shokunbi (13). The SCDBI evaluates both objective and subjective psychosocial burdens. The objective domains include the financial burden of the disease, disruption of family interactions, and disruption of routine family activities. The subjective burden assesses the caregiver's emotional response (e.g., depression, sorrow, anger, and stigma) and the family's coping ability with the disease.

The SCDBI includes 16 questions, with 3 questions each on family finances and interactions, and 5 questions each on routine family activities and caregiver coping ability. Responses are scored from 0 (never) to 3 (regularly), with higher scores indicating greater burden. Scores for each domain were categorized and interpreted as follows:

**Family finances and interactions**: 0 (no impact), 1-3 (mild impact), 4-6 (moderate impact), 7-9 (severe impact)

**Routine family activities and caregiver coping**: 0 (no impact), 1-5 (mild impact), 6-10 (moderate impact), 11-15 (severe impact)

**Data analysis**: Data were analyzed using the Statistical Package for Social Sciences (SPSS) version 24. Frequency distributions were calculated for all variables, and descriptive statistics were used to characterize the level of psychosocial burden across the different subscales.

## Results

**Socio-demographic characteristics of the caregivers**: Table 1 showed a total of 170 caregivers participated in the study. The caregivers' ages ranged from 19 to 69 years, with a mean age of 34.7 years. Females made up 83% of the participants, and 17% were male. The majority (45.9%) were between 30 and 39 years of age. One hundred and twenty-two caregivers (71.8%) had at least a secondary education. Most were traders (53.5%), followed by civil servants (27.6%). A majority (87.6%) were married, and 91.2% had only one child with SCD.

**Table 1:** Socio-demographic characteristics

Items	Frequency	Percent
Age distribution		
20-29	61	35.9
30-39	78	45.9
40-49	25	14.7
50 and above	6	3.5
Educational level		
None	19	11.1
Primary/Arabic	29	17.1
Secondary	50	29.4
Tertiary	72	42.4
Marital status		
Single	12	7.1
Married	149	87.6
Others (divorced/ separated/ widowed)	9	5.3
Sex		
Male	29	17.1
Female	141	82.9
Number of affected children		
One (1)	155	91.2
Two (2)	15	8.8
Three or more	0	0.0

**Family functioning in caregivers of children with SCD**: Table 2 showed most caregivers (68.2%) reported highly functional families (score 7-10). Twenty-three percent had moderately dysfunctional families (score 4-6), and 8.8% had severely dysfunctional families (score 0-3).

**Table 2:** Family functioning of the caregivers as assessed by family APGAR score

APGAR Score	Frequency	Percent
0-3 (severely dysfunctional)	15	8.8
4-6 (moderately dysfunctional)	39	23
7-10 (highly functional)	116	68.2
Total	170	100

### Psychosocial burden of caregivers (Table 3 & 4)

**Table 3:** frequency distribution of objective burdens (N-170)

Objective burden	Never occurs n (%)	Sometimes n (%)	Frequently n (%)	Always occur n (%)
Financial burden of caring for a child with SCD				
Income loss.	74 (43.5)	88(51.8)	6(3.5)	2(1,2)
Took loans	100(58.8)	59(34.7)	11(6.5)	0(0.0)
Expenses adversely affect family needs	85(50.0)	76(44.7)	7(4.1)	2(1.2)
Burden on routine family activities				
Neglect of other children	124(72.9)	44(25.9)	2(1.2)	0(0.0)
Difficult for child to assist at home	105(61.8)	63(37.0)	1(0.6)	1(0.6)
Difficult for child to attend school	61 (35.8)	104(61.2)	3(1.8)	2(1.2)
Disturbs activities in home	112(65.8)	53 (31.2)	3(1.8)	2(1.2)
Difficult for caregivers to engage in other activities	103(60.6)	64(37.6)	3(1.8)	0(0.0)

**Table 4:** Frequency distribution of subjective burdens (N = 170)

Subjective burden	Never occurs n (%)	Sometimes Occurs n (%)	Frequently Occurs n (%)	Always Occur n (%)
Burden on family interactions				
Child's illness cause hostility in home	139(81.8)	31(18.2)	0(0.0)	0(0.0)
Child's illness cause quarrel in family	141(82.9)	29(17.1)	0(0.0)	0(0.0)
Child's illness cause marital disharmony.	144(84.7)	26(15.3)	0(0.0)	0(0.0)
Burden on caregiver's coping ability				
Difficulty coping with child's illness	121(71.2)	47(27.6)	2(1.2)	0(0.0)
Difficulty taking responsibility for child's care	139(81.8)	31(18.2)	0(0.0)	0(0.0)
Feeling depressed about child's illness	69(40.6)	88(51.8)	10(5.8)	3(1.8)
Feeling angry because of child's illness	136(80.0)	31(18.2)	3(1.8)	0(0.0)
Feeling stigmatized because of child's illness	147(86.5)	23(13.5)	0(0.0)	0(0.0)

**Financial burden**: Ninety six caregivers (56.5%) lost income due to time spent caring for their children, and 70 caregivers (41.2%) took loans to cover medical expenses. In total, 50% reported that the illness negatively impacted their family finances.

**Routine family activities**: Approximately 64.2% of caregivers said the illness made it difficult for their child to attend school, and 39.4% said it made it hard for them to engage in other activities. In 27.1% of cases, caregivers neglected other children due to the demands of caring for their child with SCD.

**Family interactions**: The illness did not cause hostility or marital disharmony in the majority of families. Most caregivers (71.2%) had no difficulty coping with their child's illness.

**Caregiver coping**: While most caregivers (86.5%) did not feel stigmatized, 59.4% felt sorrowful or depressed about their child's illness. However, most (97.6%) did not report significant difficulties coping.

**Overall impact of the burden**: Table 5 showed caring for a child with SCD impacted family finances in 66.5%, routine family activities in 79.4%, and coping abilities in 70% of caregivers. However, there was minimal impact on family interactions (80%).

**Table 5:** Summated impact of the burden on caregivers (N = 170)

Burden domains	No impact N (%)	Mild impact N (%)	Moderate impact N (%)	Severe impact N (%)
Finance	57(33.5)	100(58.8)	12(7.1)	1(0.6)
Routine family activities	35(20.6)	128(75.3)	7(4.1)	0(0.0)
Burden on family interaction	136(80.0)	34(20.0)	0(0.0)	0(0.0)
Burden on caregiver's coping ability	51(30.0)	115(67.6)	3(1.8)	1(0.6)

## Discussion

Caring for a child with SCD is associated with both personal fulfillment and significant burdens (14). This study found that while most caregivers reported highly functional families similar to other studies in chronic conditions (15-18), the psychosocial burden was significant in areas such as family finances, routine activities, and caregiver coping.

The financial burden reported by 66.5% of caregivers mirrors findings from other studies in similar settings (19-21). The impact on routine family activities is also consistent with other research, highlighting the disruption caused by the illness(19,22,23). However, the majority of caregivers in this study reported minimal negative effects on family interactions, which is a positive indicator of strong family resilience (23,24).

In conclusion, caring for a child with sickle cell disease imposes a significant psychosocial burden, particularly in the areas of finances, routine family activities, and caregiver coping abilities. Despite this, the impact on family interactions is minimal. Most caregivers in this study reported highly functional families. Further research is needed to explore the role of family functioning in mitigating the psychosocial burden of chronic diseases like SCD.
